# Data on the individual problems and strengths scale from the systemic therapy inventory of change. Clinical samples from Norway

**DOI:** 10.1016/j.dib.2021.107577

**Published:** 2021-11-13

**Authors:** Rune Zahl-Olsen, Åshild Tellefsen Haaland, Terje Tilden

**Affiliations:** a Department of child and adolescent mental health, Sorlandet Hospital, Norway; b Modum Bad Research Institute, Norway

**Keywords:** STIC, Systemic Therapy Inventory of Change, IPS, Individual Problems and Strengths scale, EFA, Exploratory Factor Analysis, CFA, Confirmatory Factor Analysis, Systemic therapy inventory of change, STIC, Individual problems and strength, IPS, Couple and family therapy, Psychotherapy

## Abstract

These data stem from 841 clients at different couple and family therapy sites in Norway that was collected between 2010 and 2016. They all answered the Individual Problems and Strengths scale (IPS) that is a part of the Systemic Therapy Inventory of Change (STIC) system in addition to some demographic variables. In addition to the 22 items constructing the IPS scale, the data contain 14 demographic variables describing age, educational level, civil status, prior therapeutic experience, use of medicine and year of data collection. Summary statistics are provided. Male and female clients between 12 and 72 years of age answered these questions prior to or at their first session of psychotherapy. The four sites collecting the data are located at different cities in the southern part of the country and represents low and high threshold agencies.

The data can be used to test the construct validity of the measure for different populations. The data could, with a sample from the normal population, also be used for norming the scale and thus provide data to calculate cut off scores for clinical and non-clinical levels for each of the eight subscales. Further, the data could be used in combination with other measures of individual distress to test the construct validity of the scale within a Norwegian clinical sample and perhaps also within other countries.

## Specifications Table

**Table:** 

Subject	Clinical Psychology
Specific subject area	Couple and Family therapy
Type of data	Table
How data were acquired	The data were acquired through an online survey with self-evaluation questions. The survey with each item is provided in the data description section
Data format	Raw
Parameters for data collection	Participants were recruited among clients who were offered treatment at four couple and family therapy agencies. Except for the exclusion criteria for each site, accepting clients for therapy at each site, no extra exclusion criteria were applied.
Description of data collection	Data were collected as a part of a multicentre pilot study and a multicentre RCT study collecting data at every session. These data stem from the initial questionnaire. In the pilot study, some therapists asked every client to participate, others did not. In the RCT study all clients were asked to participate and each case was randomly assigned to ether treatment as usual (without the use of online feedback) or to a treatment with the use of online feedback using the STIC system (called the STIC condition) Written consent was obtained from all participants before answering the questionnaire.
Data source location	Institution 1: Modum Bad Family Unit City/Town/Region: Vikersund Country: Norway Institution 2: Department of child and adolescent mental health, Sorlandet Hospital City/Town/Region: Kristiansand Country: Norway Institution 3: The Family Counselling Unit City/Town/Region: Ålesund Country: Norway Institution 4: The Family Counselling Unit City/Town/Region: Drammen/Kongsberg Country: Norway
Data accessibility	Repository name: Mendeley Data Data identification number: https://doi.org/10.17632/fk5v8n726c.1 Direct URL to data: https://data.mendeley.com/datasets/fk5v8n726c/1

## Value of the Data

• The data can be used to the ongoing process of scale development and validation. E.g., test the construct, convergent, discriminant, divergent, factorial validity, as well as the reliability of the STIC's IPS measure for the total sample, and subsamples. It could also be used to present the level of distress, use of medication, prior experience of therapy, and educational level among clients seeking couple and family therapy in Norway.
• Researchers interested in scale development and the other psychometric characteristics, such as construct, convergent, discriminant, divergent, factorial validity, and reliability of measures of psychotherapy or in the need to compare levels of individual distress between samples can benefit from these data.
• The data could, with a sample from the normal population, be used for norming the scale and calculate cut-off scores for clinical and non-clinical levels for each subscale. Further, in combination with other measures of individual distress, the data could be used to test the construct validity of the scale.

## 1 Data Description

The data file contains raw data from 841 clients in SPSS and csv format. It has 37 variables including the ID of the participants. The Individual Problems and Strengths scale (IPS) in the Systemic Therapy Inventory of Change [1,2] consists of 22 items. The remaining variables are age, gender, therapy type, educational level, civil status, prior experience with therapy and what medications they use. In addition, data include year of data collection and what treatment condition it was collected in. Descriptive Statistics of the sample are presented in Table 1.

**Table 1:** Descriptive statistics of the sample.

	N= 841
Variable	% (N) / M [SD]
Sex: Female	51.8 (436)
Age	40.0 [8.89]
Education	
Low	51.8 (436)
Medium (Bachelor)	31.2 (262)
High (Master and PhD)	15.0 (126)
Relationship status	
Committed relationship - not married	19.3 (162)
Married	70.2 (590)
Medication	
Using medication (Some more than one medication)	32.5 (273)
Depression	13.6 (114)
Anxiety	6.9 (58)
Concentration difficulties/hyperactivity	2.6 (22)
Bipolar	3.3 (28)
Other	6.1 (51)
Prior experience with therapy	
None	25.2 (215)
Less than one year	37.2 (313)
One to three years	20.2 (170)
More than three years	16.3 (137)

The IPS scale was developed by Pinsof et al. [1] and was informed by five methodological/theoretical guidelines. First, STIC would use client self-report. Second, the data should be measured on a session-by-session basis. This second guideline implied the team to develop two versions of the IPS scale, the Initial and the Intersession STIC. The data described in this paper stems from the Initial IPS. The third guideline was to bring a multisystemic perspective to the change process. The fourth guideline was to provide a rich, and clinically relevant ‘‘picture’’ of a case and its change process. The fifth guideline refers only to the STIC Intersession and was to monitor the therapeutic alliance over the course of therapy.

The five system scales that comprise the STIC Initial were created in a four-step process [1]. The first step was when the team decided on which domains and dimensions to cover in the STIC system. They decided to include Individual Problems and Strengths (IPS), Family of Origin (FOO), Relationship with Partner (RWP), Family/Household (FH), and Child Problems and Strengths (CPS). The decision was based on what they viewed as the five most clinically relevant systems that could be consistently investigated in family, couple, and individual therapy. The second step was to ask four renowned clinicians to generate three to five items for each dimension. This step led to a long series of items for each dimension. The third step was administration of these long series of items and recruiting participants to test these items. Totally 188 clients answered to the long form of IPS. The fourth step was what they call an intermediate point in the progression from exploratory factor analysis (EFA) to confirmatory factor analysis (CFA). They began with a measurement model that specified the number of first-order factors and the items that loaded on each factor for each scale. However, the initial model failed to provide acceptable fit. After respecification they constructed a model that provided acceptable fit (RMSEA= .06 and CFI= .94) and Cronbach's alphas ranging from .54 to .89 on their dataset of 188 clients.

Items in the dataset and their answering options:

 **Client gender:** 1= Male, 2 = Female
 **Condition** refers to the study type and treatment condition the respondents were participating in. Three options are available. The first two refer to what condition (1 = STIC or 2 = TAU) in the RCT study [3] and the final, 3, signals that the data stem from the Pilot study [4].
 **Year:** Year when the client answered the questionnaire. Ranging from 2010 until 2016. Varying between 45 and 186 cases within one calendar year.
 **Therapy_type** refers to the type of therapy the case was initially identified as by the therapist. 1= Individual therapy, 2 = Couple Therapy, 3 = Family Therapy.
 **Education** refers to the educational level attained by the client. 1= Primary School, 2= High School, 3 = Education/courses higher than High School, 4 = Vocational School of education, e.g. carpenter, 5 = Single subjects at the university of university college, 6 = Bachelor's degree, 7= Master's degree, 8 = Higher than master's degree. E.g. PhD.
 **Civil_status:** Categorical variable indicating the reported civil status. 1= No relationship, 2 = Dating, 3 = Committed relationship, 4, Engaged, 5 = Married, 6 = Widow/er, 7, Separated, 8 = Divorced.
 **Medication_a-f**. Dichotomous variable indicates whether the clients currently are using medication or not for ^a)^ depression, ^b)^ anxiety, ^c)^ concentration difficulties/hyperactivity. ^d)^ bipolar, ^e)^ other, ^f)^ not applicable.
 **Therapy_experience** indicates how much prior experiences of therapy the clients have had. The categorical variable. 1 = none, 2 = 1-3 months, 3 = 4-6 months, 4 = 7-11 months, 5 = 1-3 years, 6 = more than 3 years.
 **IPS 01-28** contains the 22 variables of the STIC IPS scale. According to the developers the 22 items were theorized to tap into 8 different subscales: *Flexibility/resilience* consisted of three items: “How easy is it for you generally to overcome difficulties?”(IPS_01), “When what I'm trying doesn't work out, I can change my approach or my plans” (IPS_25) and “When I get upset, I find healthy ways to make myself feel better” (IPS_26). *Life functioning* measured consisted of two: “Performing work/school/household tasks” (IPS_02) and “Managing day-to-day life” (IPS_03). *Open expression* consisted of two items: “I can openly express my feelings” (IPS_21) and “I can speak up for myself when the situation calls for it” (IPS_22). *Self-acceptance* consisted of two items: “I can be myself in every situation” (IPS_23) and “I am comfortable with who I am” (IPS_24). *Disinhibition* consisted of three items: “Thought about seriously harming or killing someone” (IPS_11), “Had fits of rage you could not control” (IPS_12) and “Had urges or impulses that you could not control” (IPS_13). *Negative affect* consisted of six items: “Had thoughts or images over and over again that you could not get rid of” (IPS_05), “Felt tense or anxious” (IPS_06), “Felt sad most of the day” (IPS_07), “Thought about ending your life” (IPS_08), “Felt hopeless about the future” (IPS_09) and “Not enjoyed things as much as you used to” (IPS_10). *Self-misunderstanding* consisted of two: ”I don't understand why I do the things I do” (IPS_27) and “It's tough for me to know what I'm feeling” (IPS_28). *Substance abuse* consisted of two items: “Drank too much alcohol” (IPS_14) and “Used illegal drugs/misused prescribed medication” (IPS_15).

• The answering options for IPS_01 are: 1 = Very hard; I get very down and recover very slowly if at all, 2 = Fairly hard; I get pretty down and it takes a long time to recover, 3 = So-so; I often struggle for a while, but get better eventually, 4 = Fairly easy; things don't get me down too much, 5 = Quite easy; I bounce back quickly
• The answering options for IPS_02 and 03 are: 1= Quite poorly, 2 = Fairly poorly, 3 = So-so, 4 = Fairly well, 5 = Quite well.
• The answering options for IPS_05 until 15 are: 1 = All of the time, 2 = Often, 3 = Sometimes, 4 = Rarely, 5 = Not at all/never.
• The answering options for IPS_21 until 28 are: 1 = Not at all true, 2 = Mostly not true, 3 = Somewhat true, 4 = Mostly true, 5 = Very true.
• IPS_04, 16, 17, 18, 19, 20 are not included in the IPS scale.

The sample's level of distress on the IPS items is presented in Table 2.

**Table 2:** Descriptive Statistics of the sample on the IPS items.

	N	Min	Max	Mean	SD
IPS_01	835	1	5	3,19	0,81
IPS_02	836	1	5	3,38	0,97
IPS_03	836	1	5	3,31	0,85
IPS_05	835	1	5	3,41	1,23
IPS_06	836	1	5	2,78	1,01
IPS_07	837	1	5	3,25	1,01
IPS_08	836	2	5	4,64	0,71
IPS_09	836	1	5	3,30	1,05
IPS_10	836	1	5	3,24	1,06
IPS_11	837	2	5	4,93	0,32
IPS_12	836	2	5	4,17	0,91
IPS_13	836	1	5	4,44	0,85
IPS_14	837	2	5	4,67	0,61
IPS_15	837	2	5	4,95	0,26
IPS_21	836	1	5	3,47	1,24
IPS_22	836	1	5	3,83	1,09
IPS_23	836	1	5	3,11	1,20
IPS_24	835	1	5	3,17	1,30
IPS_25	835	1	5	3,80	0,96
IPS_26	835	1	5	3,45	1,13
IPS_27	836	1	5	3,62	1,22
IPS_28	836	1	5	3,39	1,29

## 2 Experimental Design, Materials and Methods

The data were acquired by the use of the STIC online platform that was provided by the Family Institute at the North-Western University in Evanston and Chicago, Illinois, USA. The data - the clients’ answers - were stored in a secure server provided by the Family Institute. The data were sent to us in raw format. To get all items presented as “higher is better”, some of the IPS items had to be recoded, i.e., inversed so that 5 becomes 1, 4 becomes 2 and so on. This recoding applies to IPS_05-IPS_15 and IPS_27-28. In the dataset, all those items are labelled “HBRecode”.

To produce Table 1, item responses to Education were grouped to Low (item response 1-5), Medium (item response 6) and High (item response 7 and 8). Prior experience with therapy was also grouped into none (item response 1), less than one year (item response 2-4), one to three years (item response 5), and more than three years (item response 6).

## Ethics Statement

Informed consent was obtained for experimentation with human subjects and approval was given by the regional ethical committee in southern Norway (2009/927, REK sør-øst). For minors to participate their legal guardians had to sign an informed consent regarding their minors’ participation.

## CRediT authorship contribution statement

**Rune Zahl-Olsen:** Project administration, Data curation, Writing – original draft, Methodology. **Åshild Tellefsen Haaland:** Project administration, Data curation, Writing – review & editing. **Terje Tilden:** Investigation, Project administration, Data curation, Writing – review & editing.

## Declaration of Competing Interest

The authors declare that they have no known competing financial interests or personal relationships that have or could be perceived to have influenced the work reported in this article.
